# Pamidronate for pain in adult chronic nonbacterial osteitis: protocol of a randomized, double-blind, placebo-controlled trial

**DOI:** 10.1093/jbmrpl/ziae114

**Published:** 2024-08-27

**Authors:** Anne T Leerling, Ana Navas Cañete, Frits Smit, Neveen A T Hamdy, Alina van de Burgt, Natasha M Appelman-Dijkstra, Olaf M Dekkers, Elizabeth M Winter

**Affiliations:** Department of Internal Medicine, Division of Endocrinology, Leiden University Medical Center, Leiden, 2333 ZA, the Netherlands; Center for Bone Quality, Leiden University Medical Center, Leiden, 2333 ZA, the Netherlands; Department of Clinical Epidemiology, Leiden University Medical Center, Leiden, 2333 ZA, the Netherlands; Center for Bone Quality, Leiden University Medical Center, Leiden, 2333 ZA, the Netherlands; Department of Radiology, section of Nuclear Medicine, Leiden University Medical Center, Leiden, ZA 2333, the Netherlands; Center for Bone Quality, Leiden University Medical Center, Leiden, 2333 ZA, the Netherlands; Department of Radiology, section of Nuclear Medicine, Leiden University Medical Center, Leiden, ZA 2333, the Netherlands; Department of Nuclear Medicine, Alrijne Hospital, Leiderdorp, 2353 GA, the Netherlands; Department of Internal Medicine, Division of Endocrinology, Leiden University Medical Center, Leiden, 2333 ZA, the Netherlands; Center for Bone Quality, Leiden University Medical Center, Leiden, 2333 ZA, the Netherlands; Department of Nuclear Medicine, Alrijne Hospital, Leiderdorp, 2353 GA, the Netherlands; Department of Internal Medicine, Division of Endocrinology, Leiden University Medical Center, Leiden, 2333 ZA, the Netherlands; Center for Bone Quality, Leiden University Medical Center, Leiden, 2333 ZA, the Netherlands; Department of Internal Medicine, Division of Endocrinology, Leiden University Medical Center, Leiden, 2333 ZA, the Netherlands; Department of Clinical Epidemiology, Leiden University Medical Center, Leiden, 2333 ZA, the Netherlands; Department of Internal Medicine, Division of Endocrinology, Leiden University Medical Center, Leiden, 2333 ZA, the Netherlands; Center for Bone Quality, Leiden University Medical Center, Leiden, 2333 ZA, the Netherlands

**Keywords:** osteitis, osteomyelitis, bisphosphonates, pamidronate, rare diseases, CNO, CRMO, sapho, pain, [^18^f]naf-pet/ct, imaging, clinical trial

## Abstract

Chronic nonbacterial osteitis (CNO) is a rare auto-inflammatory bone disease affecting children and adults. Adult CNO is characterized by painful bone lesions, primarily of the anterior chest wall. There is no approved therapy for adult CNO. Current off-label treatments include intravenous bisphosphonates, which have been shown to alleviate pain through decreasing bone turnover. However, no adequately powered randomized controlled trials (RCTs) have been conducted. This double-blind, placebo-controlled RCT investigates the efficacy of intravenous pamidronate to decrease bone pain in adult CNO patients. Recruiting at the Dutch national referral center for CNO, adult patients with persistent bone pain despite non-steroidal anti-inflammatory drugs, or optionally other standard-of-care treatments are randomized to receive two courses of intravenous pamidronate (at 0 and 3 mo, 30 mg daily, on 3 consecutive d) or placebo. From 6 mo onwards, all patients receive open-label pamidronate for another two courses. The primary outcome is change in score for maximum pain from 0 to 6 mo. Secondary outcomes include change in quantitative intralesional bone turnover as measured on sodium-fluoride positron emission computed tomography ([^18^F]NaF-PET/CT), inflammation markers, shoulder function, general health, quality of life, fatigue, physical, and work activity. The pamidronate for pain in adult chronic nonbacterial osteitis trial addresses the need for evidence-based treatments in adult CNO. Results will directly impact daily clinical practice, either validating the use of intravenous pamidronate in CNO at the dose used in this trial or prompting the search for alternative regimens or agents. This trial was registered in EudraCT (reference 2020-001068-27) and the Dutch Trial Register (reference NL68020.058.20).

## Introduction

Chronic nonbacterial osteitis (CNO) is a rare autoinflammatory bone disease occurring in children and adults. In adults, the group involved in this trial, CNO primarily affects the axial skeleton, specifically the anterior chest wall. The spine, and to a lesser extent the mandible, and appendicular skeleton may also be involved.^1^^,^^2^ The precise pathophysiology of the disorder remains to be fully elucidated, although it has been shown to involve systemic cytokine dysregulation, leading to chronic bone inflammation and locally increased bone turnover.^3^ Over time, this results in erosions, degeneration, and excess bone formation in the form of hyperostosis and soft tissue calcification.^4^^,^^5^ Patients experience swelling and pain at the site of bone lesions, along with compromised functioning of adjacent joints. Therefore, CNO frequently causes decreased quality of life and work absence.^6^^,^^7^ While isolated bone involvement is most commonly observed, CNO patients may present with additional inflammatory features such as palmoplantar pustulosis, psoriasis, or arthritis.^2^

Clinical management of CNO is challenging, as there are neither evidence-based or standard therapies nor validated disease activity measures, resulting in significant variation in care world-wide.^8^ Current treatment options for both pediatric and adult CNO include non-steroidal anti-inflammatory drugs (NSAIDs) or cyclooxygenase 2 inhibitors (COXIBs), and in patients with persistent pain despite these agents, intravenous bisphosphonates or tumor necrosis factor alpha inhibitors (TNFi) may be administered.^2^^,^^8^^,^^9^ For pediatric CNO, consensus treatment plans centered around these agents have already been established,^9^ and for adult CNO, these are in development.^10^ However, randomized controlled trials (RCTs) are lacking for all treatments; their use in all age groups are primarily based on observational data from small cohorts and case series.

**Figure 1:**
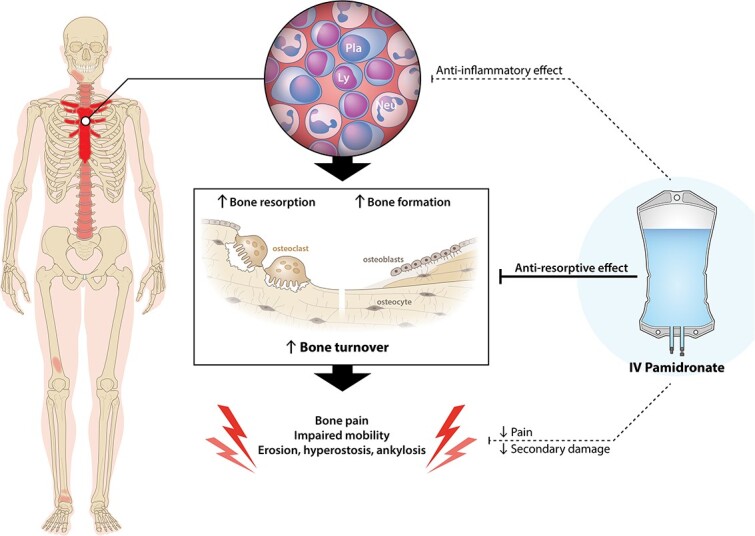
Hypothesized working mechanism of pamidronate in chronic nonbacterial osteitis (CNO). Legend: IV: Intravenous, Ly: Lymphocyte, Neu: Neutrophil, Pla: Plasma cell.

Bisphosphonates are antiresorptive agents that are selectively taken up by bone at sites of increased bone turnover. Here, they prompt function loss of osteoclasts, thereby exerting their anti-resorptive effect.^11^^,^^12^ The rationale for using bisphosphonates in both adult and pediatric CNO is based on the fact that nearly all patients with clinically active disease demonstrate increased bone turnover at affected sites, which in adults is mostly visualized through increased isotope uptake on nuclear imaging (see Figure 1).^2^ Increased bone turnover has been associated with pain in other metabolic bone diseases and is the driver of long-term structural tissue changes.^13^^,^^14^ It is thus hypothesized that bisphosphonates alleviate pain and prevent disease progression by decreasing local turnover in CNO as well.^15^ Besides, bisphosphonates may also target the inflammation that triggers the increase in bone turnover in CNO, by inhibiting the Farnesyl Pyrophosphate (FPP) synthase enzyme, which is essential for the survival not only of osteoclasts but also of FPP-dependent macrophages.^16^ Indeed, bisphosphonates, specifically pamidronate, have been shown to partially inhibit lymphocyte proliferation, monocyte/lymphocyte interaction, and gamma/delta T-cell numbers.^17^

The theoretical efficacy of bisphosphonates in CNO is supported by observational data, showing how bisphosphonates, mainly intravenous pamidronate, lead to a reduction in pain and radiologic signs of disease activity in adults and children.^18^^-^^28^ Similar positive results were found in a first small randomized, double-blind, placebo-controlled *pilot* trial comparing pamidronate to placebo in 12 adult CNO patients.^29^ Adequately powered trials are essential to infer a causal relationship regarding the efficacy of pamidronate to reduce pain in CNO, elucidate the underlying mechanisms, gain deeper understanding of the optimal treatment regimen, gather information on safety, and identify predictors of therapy response. Hence, we conceptualized and launched an investigator-initiated RCT PAMidronate for pain in adult Chronic Nonbacterial Osteitis (PAM-CNO). The design and conduct of this RCT presents challenges, due to the rarity of the disease, the absence of a clear disease definition, and limited understanding of relevant outcome measures. We therefore report its protocol, including the methodological obstacles characteristic of doing research in rare and poorly defined diseases such as CNO.

## Objectives

The primary objective of the PAM-CNO trial is to investigate the efficacy of intravenous pamidronate in reducing pain in adult CNO patients. Secondary objectives are to evaluate the effect of pamidronate on (1) a variety of patient-reported outcome measures (PROMs), (2) biochemical and immunological indices relevant to CNO, and (3) radiological parameters of disease activity and severity. Additional objectives are to evaluate baseline predictors of treatment response, to elucidate mechanisms by which pamidronate may reduce pain in CNO, and cost-effectiveness.

## Materials and methods

### Trial setting and design

**Figure 2:**
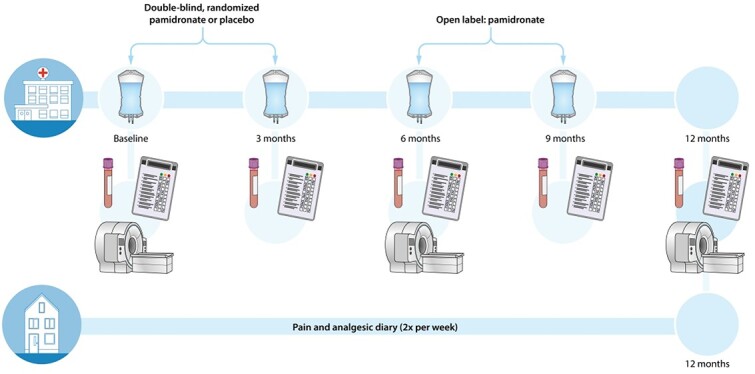
Study structure of the PAM-CNO trial.

This is a single-center, superiority trial conducted at the Centre for Bone Quality of the Leiden University Medical Center (LUMC), which is the Dutch national expertise and referral center for CNO. The trial starts with a 6-mo double-blind, randomized, placebo-controlled phase with 1:1 allocation of pamidronate or placebo (see Figure 2). Double-blind placebo-control was deemed necessary to prevent reporting bias because the trial’s primary outcome is patient-reported pain, which is susceptible to a placebo-effect. The 6-mo treatment duration is based on observational data from our center, where patients typically receive treatment for 6 mo or 1 yr (2 or 4 infusion cycles, administered in 3-mo intervals aligning with the physiological cycle of bone turnover). In consultation with representatives of the Dutch CNO patient association, it was agreed that 6 mo period of potential placebo treatment was acceptable if followed by the open-label extension. Therefore, the 6-mo randomized phase is followed by a 6-mo open-label phase.

### Eligibility criteria

Patients ≥18 yr of age with an established diagnosis of CNO are the population base of this study. In the absence of validated classification criteria for CNO, the primary diagnostic criterion is sterile bone inflammation at one or more skeletal sites in the anterior chest wall, with or without involvement of other skeletal sites. This is identified based on clinical features (inflammatory bone pain and possibly signs of inflammation on physical exam), biochemical markers (mild to moderate increase in inflammation markers, though often these are within normal range), and imaging findings (bone marrow edema, sclerosis, hyperostosis, erosions, ligament ossification, ankylosis of joints, and increased isotope uptake on nuclear imaging). If a bone biopsy is performed, histopathology compatible with sterile bone inflammation includes nonspecific inflammation and increased bone turnover. Sterile bone inflammation, and herewith diagnosis of CNO, is confirmed through a multidisciplinary, integrated assessment. Of note, this definition of sterile bone inflammation/CNO means that patients can have varying skeletal distribution patterns and may or may not have additional (extra)skeletal features such as arthritis, enthesitis, sacroiliitis, palmoplantar pustulosis, psoriasis, or severe acne.

Further inclusion criteria are as follows:

1. Score for maximal pain (concerning bone pain at lesion sites over the past week) ≥6/10 as measured by Brief Pain Inventory (BPI).^30^
2. Insufficient response to at least one NSAID/COXIB at maximum approved and tolerated dosage, intolerance or contraindication to these agents, or indication for direct second-line treatment due to active vertebral lesions.
3. A washout period of prior bisphosphonates (6 mo since last administration).

Exclusion criteria are as follows:

1. <18 yr of age.
2. Active pregnancy wish, active pregnancy, or nursing.
3. Generalized pain without pain at the site of CNO lesions.
4. Bisphosphonate allergy.
5. Estimated glomerular filtration rate < 30 ml/min.
6. Uncontrolled endocrine comorbidities, active cancer (treatment), or other severe comorbidity preventing the attendance of study visits.
7. Severe language barrier preventing the completion of questionnaires.
8. Mental disability.

Of note, eligible patients receive supplementation before study inclusion if serum 25-hydroxy vitamin D is <50 nmol/L. Eligible patients with poor dental hygiene are included only after consultation and approval from an oromaxillary surgeon, due to the small risk of osteonecrosis of the jaw (ONJ) associated with bisphosphonate treatment.^31^

### Interventions

Pamidronate is administered at 0 and 3 mo, an interval that is common practice due to the known pharmacodynamic and pharmacokinetic properties of this agent and which aligns with the physiological cycle of bone turnover.^12^ In total, 90 mg is administered over the course of 3 d of 30 mg each. The splitting of the total dose is done to get optimal uptake at sites of increased bone turnover, as pamidronate is quickly cleared from the circulation.^12^ The 30-mg dose is diluted in 250 ml 0.9% saline, administered intravenously in 2 h. Placebo is administered as 250 ml of saline 0.9% at the same speed, also over 3 consecutive d. Randomization, preparation, and (blind) labeling of pamidronate and placebo are done by the certified LUMC pharmacy according to the Good Manufacturing Practice-guidelines.

### Concomitant care and deviations from allocated interventions

For concomitant care, both the placebo and intervention arms allow additional NSAIDs/COXIBs at stable dosages. Intake of NSAIDs/COXIBs is monitored by the research team through a diary completed by the patient at 2 fixed d per wk (Wednesday and Saturday). NSAIDs/COXIBs may be decreased or discontinued at any point if pain levels allow. Principally, NSAIDs/COXIBs are not increased, except in cases of unacceptable persistent pain, where they are considered escape medication.

Since CNO patients may suffer from other (extra)skeletal manifestations, it is anticipated that some patients may be taking other standard-of-care anti-inflammatory treatments, such as methotrexate for arthritis or biologic disease-modifying anti-rheumatic drugs (biologic DMARDs) for psoriasis. To limit undertreatment of such patients, all standard-of-care treatments may be continued throughout the trial provided they are taken at stable dosage. Importantly, the main inclusion criterion of a ≥6/10 score for maximal bone pain must be met despite use of these standard-of-care treatments, as evidence for insufficient control of osteitis. Also, if standard-of-care treatments have been discontinued or changed shortly before anticipated inclusion, a washout period is installed as recommended based on half-life by the pharmacology department (eg, 1-4.5 mo for TNFi, depending on the agent and administration route). During the trial, standard-of-care anti-inflammatory treatments are not newly started or increased unless urgently needed. Patients are permitted to continue, start, or discontinue physical therapy as needed.

### Outcomes and statistical analyses

The primary outcome of this trial is score of maximal pain on BPI (numerical rating scale 0-10) measured at the end of the 6-mo randomized phase. We will compare the difference between groups by generalized linear mixed model (GLMM) with analysis of covariance (ANCOVA) to account for baseline scores. The primary analysis will be intention-to-treat (see Table 1 and Figure 2). Secondary outcomes include a range of patient-reported,^32^^-^^40^ biochemical, and radiological outcomes, as well as their intercorrelation, baseline factors associated with treatment response,^41^^,^^42^ and cost-effectiveness^43^ (see Table 1 for measurement instruments, statistical analyses, and timepoints). Sensitivity analyses include a per-protocol and an as-treated analysis of the primary outcome. Missing data on the outcomes not evaluated by GLMM are imputed and handled through maximum likelihood techniques. No interim analyses are conducted.

**Table 1:** Primary and secondary outcomes of the PAM-CNO trial.

	**Outcome**	**Instrument**	**Timepoint of collection**	**Statistical analysis**	**Timepoint of analysis**
**Primary**	Score for maximal pain at CNO lesion sites	BPI	0, 3, 6, 9 and 12 mo	GLMM with ANCOVA	6 mo
**Secondary**	Score for minimal, average, and activity-related pain at CNO lesion sites	BPI	0, 3, 6, 9 and 12 mo	GLMM with ANCOVA	6 and 12 mo
**PROMs**	Shoulder function	SFA			
		SRQ			
	General health	SF-36			
	Quality of Life	EQ-5D			
	Work activity	Work Activity Score			
	Physical activity	IPAQ			
	Fatigue	MVI-20			
	Patient global	PGIC			
	Burden of a relative	Carer-QoL			
	Pain diary	NRS 0-10 for pain	2 d per wk, for entire 1-yr trial span	GLMM	6 and 12 mo
	Achievement of mild pain, as defined by ≤4/10 for maximal pain on BPI	BPI	0, 3, 6, 9, and 12 mo	Chi-square between groups, reported as odds ratio with 95% confidence interval	3, 6, and 12 mo
	Achievement of ≥50% reduction in maximal pain on BPI	BPI	0, 3, 6, 9, and 12 mo		
	% of patients with reduction in standard dose of NSAIDs/COXIBs	-	3, 6, and 12 mo		
**Biochemical**	ESR, CRP	Standard laboratory evaluation	0, 3, 6, 9, and 12 mo	GLMM with ANCOVA	6 and 12 mo
	Pro- and anti-inflammatory cytokines	ELISA			
	Bone markers	ELISA			
	mRNA expression	MLPA	0, 6, and 12 mo		
	T-lymphocyte counts	FACS			
**Radiological**	Progression or regression of either sclerosis, hyperostosis, erosions, ankylosis, or soft tissue calcification	[^18^F]NaF-PET/CT	0, 6, and 12 mo	Chi-square between groups, reported as odds ratio with 95% confidence interval	6 and 12 mo
	Quantitative isotope uptake in CNO-lesions by SUV_max_			GLMM with ANCOVA	6 and 12 mo
**Correlations**	SUV_max_ with maximal and average pain	[^18^F]NaF-PET/CT and BPI	0, 6, and 12 mo	Pearson’s R or Spearman’s Rho as appropriate	0, 6, and 12 mo
	SUV_max_ with ESR and CRP	[^18^F]NaF-PET/CT and standard laboratory evaluation	0, 6, and 12 mo		
**Predictors of treatment response**	Diagnostic delay (yr)	-	0 mo	GLMM in pamidronate group, with maximal pain score on BPI as outcome	6 mo
	Baseline lesional isotope uptake (SUV_max_)	[^18^F]NaF-PET/CT	0 mo		
	Degree of central sensitization	CSI	0, 6, and 12 mo		
	Degree of neuropathic pain	PAIN-DETECT	0, 6, and 12 mo		
	Baseline presence of erosions, hyperostosis, and/or ankylosis	[^18^F]NaF-PET/CT	0 mo		
	Baseline presence of elevated ESR and/or CRP	-	0 mo		
**Cost-effectiveness**	Trial-based cost-utility from a societal perspective in accordance with the Dutch health-economics guidelines	IPCQ	0, 3, 6, 9, and 12 mo	Trial-based cost-utility analysis with costs-per-quality adjusted life yr, based on net-benefit analysis and friction-cost method.	6 and 12 mo (extrapolated to life-long discounted time horizon)

Abbreviations: ANCOVA; Analysis of Covariance, [18F]NaF-PET/CT; Sodium-fluoride positron emission tomography, BPI; Brief Pain Inventory, CarerQoL; Care-related Quality of Life Instrument, CNO; Chronic Nonbacterial Osteitis, COXIBs; Cyclooxygenase 2 Inhibitors, CSI; Central Sensitization Inventory, FACS; Fluorescence Activated Cell Sorting, ELISA; enzyme-linked immunosorbent assay, EQ-5D; EuroQol 5-Dimension, ESR; Erythrocyte Sedimentation Rate; GLMM; Generalized Linear Mixed Model, IPAQ; International Physical Activity Questionnaire, MLPA; Multiplex Ligation-dependent Probe Amplification, MVI-20; Multidimensional Fatigue Inventory-20, NRS; Numerical Rating Scale, NSAIDs; Non-Steroidal Anti-Inflammatory Drugs, PGIC; Patient Global Impression of Change, SF-36; Short Form Health Survey, SRQ; Shoulder Rating Questionnaire, SUV_max_; Standardized Maximum Uptake Value.

### Sample size

Based on our observational data on adult CNO patients with a maximum pain score of ≥6/10 treated with intravenous pamidronate, BPI score for maximal pain is expected to decrease from a mean of 8 with standard deviation (±) of 2 to a mean of 6 ± 2 in the active treatment arm. In rheumatoid arthritis, the placebo effect on pain has been estimated at 16-22 at a 100-mm scale.^44^ However, because evidence-based treatments for CNO are entirely lacking—potentially reducing patient confidence in any given treatment—and many patients present with advanced disease due to diagnostic delays, we anticipate a smaller effect in the placebo arm of this trial, with pain levels decreasing from 8 ± 2 to 7.5 ± 2. We use a power of 80% and an alpha of 0.05. As the primary analysis is done with ANCOVA, adjustment for correlation between baseline and follow-up scores was done (Pearson’s correlation coefficient calculated at 0.479), with a required sample size of 35 patients per arm (total *n* = 70).^45^ In case of screen failure or drop-out before the 6-mo primary endpoint, patients will be replaced to meet the required sample of 70.

### Screening, recruitment, and informed consent

Patients are recruited directly at the national referral center for CNO. Awareness of the trial amongst patients is ensured by collaborating with the Dutch CNO patient association. Patients are identified at the outpatient clinic by their treating physician and referred for screening by the research team. Screening includes the verification of all specified in- and exclusion criteria. Written informed consent is obtained after a structured interview by EMW, NMA, or ATL, all authorized according to Good Clinical Practice-guidelines. Upon consent of the patient, a relative is parallelly recruited to fill out a questionnaire on carer burden (see Table 1). Recruitment has started in December 2020 and is anticipated to be completed in May 2025.

### Trial workplan and data collection

Visits are scheduled at 0, 3, 6, 9, and 12 mo (see Figure 2), with pamidronate or placebo administered at 0 and 3 mo, and open-label pamidronate administered at 6 and 9 mo. Visit 12 is an outpatient consultation without infusions.

At every visit, patients complete a set of questionnaires (see Table 1). BPI and shoulder function assessment (SFA) are filled out in writing on paper as part of standard care [recorded in the electronic health record and case report form (CRF)]. All other questionnaires are digitally completed by the patient in the CRF. A relative, if included, digitally completes the questionnaire on carer burden at every timepoint as well. Patients are requested to keep a digital pain and fatigue diary (NRS 0-10, range from pain/fatigue to 10 for worst pain/fatigue) and record their analgesic use for 2 fixed d each wk (Wednesday and Saturday) throughout the entire trial.

Other CRF-entries include the recording of medication changes, adverse events, physical examination, and fasting-state laboratory investigations (complete blood and differential count, CRP, ESR, kidney function, calcium/albumin, phosphate, alkaline phosphatase, gammaGT, bone turnover markers C-terminal telopeptide of type I collagen and Procollagen type I N-terminal propeptide, parathyroid hormone, 25-hydroxy vitamin D).

Serum and plasm are stored within 2 h after specimen withdrawal at −80 °C, for future batch analysis of inflammatory markers and additional bone markers with enzyme-linked immunosorbent assay. At 0, 6, and 12 mo, additional specimens are collected for isolation of mRNA and peripheral blood mononuclear cells (harvested <2 h after blood withdrawal and stored in liquid nitrogen). In batch, Multiplex Ligation-dependent Probe Amplification-assay characterizing mRNA expression will be performed, after which specific T-cell subsets of interest will be counted with Fluorescence Activated Cell Sorting, including Th-17 and γδ T-cells.

For imaging evaluation, we selected sodium fluoride positron emission tomography ([^18^F]NaF-PET/CT), because it offers several advantages compared with other common techniques such as magnetic resonance imaging (MRI) and bone scintigraphy using technetium-99 m radio-labeled hydroxymethylene diphosphonate ([^99^mTc]Tc-HDP). [^18^F]NaF-PET/CT offers a higher spatial resolution and is therefore more sensitive than MRI to visualize structural features such as sclerosis and hyperostosis, especially in the region of anterior chest wall.^29^ Compared with [^99^mTc]Tc-HDP, [^18^F]NaF-PET/CT allows for shorter scanning time lower radiation exposure. Finally, [^18^F]NaF-PET/CT provides quantitative data on bone turnover that correlate with clinical disease activity in other metabolic bone diseases. We recently demonstrated that these quantitative data can be generated for CNO as well and may candidate as disease activity imaging biomarkers.^46^^-^^48^ Quantitative [^18^F]NaF-PET/CT data may be preferred over other imaging biomarkers, such as [^99^mTc]Tc-HDP-based isotope uptake, which has been shown to have an imprinting pattern.^49^ Additionally, it may be favored over bone marrow edema detection on MRI, as edema cannot be adequately detected or quantified in sclerotic bone.^50^ [^18^F]NaF-PET/CT is performed at 0, 6, and 12 mo, using a 5-ring Discovery MI PET/CT machine (GE Healthcare, Chicago, IL, USA). Data are acquired ~30 min after intravenous Na18F administration at a dose of around 1-MBq/kg body weight. A single experienced nuclear physician and radiologist assess all [^18^F]NaF-PET/CT scans using a standardized reporting format, with consensus-based assessment in cases of disagreement.^5^^,^^46^ Each potential lesion site is evaluated for sclerosis, hyperostosis, erosions, ankylosis, and [^18^F]NaF-uptake. Quantitative [^18^F]NaF-uptake is determined by drawing volumes of interest in CNO lesions and reference bone identified by CT.

### Blinding

Patients, other care providers, outcome assessors, and data analysts are blinded for intervention allocation; randomization is done in 1:1 ratio, in a 2-4-6 block structure using Castor EDC. The code is kept under seal by the LUMC pharmacy, only to be broken in the event of a medical emergency.

### Data management

All data are recorded using Castor EDC’s CRF. Biological specimens are collected under the unique study number without patient identifiable information. Handling of personal data is done in compliance with the General Data Protection Act. Data are stored for 25 yr.

### Oversight and monitoring

The trial is led on day-to-day basis by the coordinating and principal investigator. Progress and issues are discussed in periodic meetings, including representatives of the radiology and nuclear medicine department to address organizational and scientific aspects of the [^18^F]NaF-PET/CT scans as needed. Based on the risk-classification set by the Dutch Committee of Human-involved Research, no data and safety monitoring committee is installed and in-house auditing is performed on annual basis. Substantial amendments are notified to the institutional review board and to the competent authority.

### Adverse event reporting and harms

Adverse effects of pamidronate are well-known, due to its widespread use in bone disease over the past decades.^51^ The only expected adverse effect of pamidronate is acute phase reaction, especially after a first-ever infusion, which is generally manageable with paracetamol. ONJ and atypical femoral fractures have been reported as rare but serious adverse effects of pamidronate. However, these are not expected based on this trial’s dose and treatment duration and have never been reported in this patient population so far. As dental procedures increase the risk of ONJ, patients with poor oral hygiene are enrolled only after consultation with an oral and maxillofacial surgeon. All patients are requested to have dental examination twice a year, and a letter informing on the use of bisphosphonates is sent to the treating dentist upon trial inclusion. All adverse events reported spontaneously by the patient or observed by the research team are recorded. All serious adverse events are reported adhering to the Dutch Committee of Human-involved Research guidelines. An annual safety report is submitted at the institutional review board of the LUMC and Dutch, the Central Committee on Research Involving Human Subjects as well.

### Dissemination plans

Data obtained from this study will be unreservedly disclosed and published. Both positive and negative results of this study will be submitted for publication to peer-reviewed scientific journals.

## Discussion

The PAM-CNO trial marks a significant milestone as a first powered double-blind, randomized, placebo-controlled trial investigating the effectiveness of intravenous pamidronate in 3-mo cycles to reduce pain in adults with CNO. As current treatment for CNO relies on expert opinion and scarce observational data, the trial’s outcomes are expected to have direct clinical impact, either validating the use of pamidronate or prompting the search for other effective treatments.^52^

Designing and conducting an RCT for a rare and poorly characterized disease such as CNO presented multiple challenges. First, the considerable worldwide variation in disease definition and the absence of validated classification criteria made it difficult to establish inclusion criteria. Our current inclusion criteria are derived from a structured multidisciplinary assessment that combines typical clinical, biochemical, imaging, and—if bone biopsy is performed—histopathological characteristics. As a national expertise center for CNO, LUMC handles high patient volumes, providing us with extensive diagnostic experience. We therefore anticipate that our study population will be representative of CNO, but acknowledge the importance of developing classification criteria to improve the external validity of future trials.

Recruitment represents a second challenge in trial conduct. The required sample size of 70 patients was initially expected to be recruited over the course of 18 mo starting in December 2020, but this was not achieved due to the rarity of the disease and external factors (such as the COVID-19 pandemic limiting outpatient contacts). While the possibility of expanding to a multicenter trial was considered, this option was rejected due to the variation in disease definition and diagnostic approach, and the limited availability of laboratory infrastructure and [^18^F]NaF-PET/CT at other centers. The recruitment period was extended, and with 61/70 patients included at current, recruitment is anticipated to be completed in the first half of 2025.

A third challenge encountered in this trial was the choice for the primary outcome of patient-reported pain. Ideally, we would use a composite score that includes patient-reported outcomes such as pain and objective measures of inflammatory activity, reflecting both patient relevance and the biological mechanisms of treatment. However, for CNO, no objective markers of inflammatory activity are currently proven. Systemic inflammation markers are suboptimal indicators of disease activity due to their frequently normal levels at diagnosis,^2^ and the same holds for radiologic signs of disease activity such as increased isotope uptake, since it has been shown to persist despite evident clinical improvement, which may prompt overtreatment.^49^ Consequently, we use patient-reported pain at the site of CNO bone lesions as the primary outcome of this trial. While this is most relevant for patients, it may be modulated via different mechanisms which may complicate interpretation of the results. Therefore, patients are clearly instructed to differentiate and score the nociceptive inflammatory bone pain.^53^ Additionally, neuropathic and nociplastic pain are measured with standardized questionnaires to explore their association with treatment response. Recognizing that radiologic signs of disease activity in CNO need to be further investigated, this trial includes quantified uptake on [^18^F]NaF-PET/CT in the secondary analyses. This imaging modality is sensitive for bone pathology and yields quantitative parameters of bone turnover, which have shown to be elevated in CNO lesions and correlate with ESR.^46^ The longitudinal data on quantified [^18^F]NaF uptake from this trial will further elucidate the working mechanism of pamidronate, revealing whether pamidronate can quantitatively reduce bone turnover, and whether a decrease is associated with a reduction in pain.

A final essential consideration of the PAM-CNO trial will be whether its results will be applicable to the pediatric CNO population as well. From a clinical perspective, there are similar observational data supporting the use of pamidronate in pediatric CNO, and this trial may reinforce the clinical body of evidence.^25^^,^^54^ From a pathophysiological perspective, it appears that adult and pediatric CNO may share common auto-inflammatory mechanisms, which may lend further credence to the use of pamidronate in both age groups.^55^ Therefore, results of the PAM-CNO trial may extrapolate to pediatric CNO, especially since bisphosphonates have shown to be safe and well-tolerated in children too.^56^

In conclusion, this first RCT on the effectiveness of pamidronate in adult patients with CNO is expected to have a significant impact, as there is currently no established treatment for this rare condition. Should pamidronate be found effective for pain control, future research should explore the optimal dosage and treatment schedule, and identify specific patient subgroups which may benefit the most. Also, pamidronate should be compared against TNFi which is commonly also used as second-line treatment in adult CNO. Additionally, this study will provide useful information on whether quantified [^18^F]NaF uptake can serve as an indicator of disease activity and treatment response in adult CNO. If the data support this idea, subsequent studies may consider this parameter when developing composite scores for disease activity in adult CNO.

## Data Availability

The full protocol, participant-level data and statistical code are available upon reasonable request.

## Abbreviations

ANCOVA—Analysis of Covariance; [^18^F]NaF-PET/CT—Sodium-fluoride positron emission tomography; BPI—Brief Pain Inventory; CarerQoL—Care-related Quality of Life Instrument; CNO—Chronic Nonbacterial Osteitis; COXIBs—Cyclooxygenase 2 Inhibitors; CRF—Case Report Forms; CSI—Central Sensitization Inventory; DMARDs—Disease-Modifying Anti-Rheumatic Drugs; ELISA—enzyme-linked immunosorbent assay; EQ-5D—EuroQol 5-Dimension; ESR—Erythrocyte Sedimentation Rate; FACS—Fluorescence Activated Cell Sorting; FPP—Farnesyl Pyrophosphate; GCP—Good Clinical Practice; GLMM—Generalized Linear Mixed Model; GMP—Good Manufacturing Practice; IPAQ—International Physical Activity Questionnaire; MLPA—Multiplex Ligation-dependent Probe Amplification; MVI-20—Multidimensional Fatigue Inventory-20; NRS—Numerical Rating Scale; NSAIDs—Non-Steroidal Anti-Inflammatory Drugs; PGIC—Patient Global Impression of Change; QALY—Quality Adjusted Life Year; RCTs—Randomized, Placebo-Controlled Trials; SF-36—Short Form Health Survey; SFA—Shoulder Function Assessment; SRQ—Shoulder Rating Questionnaire; SUV_max_—Standardized Maximum Uptake Value; TNFi—Tumor Necrosis Factor Alpha Inhibitors.
